# Authors' reply: Meta‐analysis of goal‐directed fluid therapy using transoesophageal Doppler in patients undergoing elective colorectal surgery

**DOI:** 10.1002/bjs5.50228

**Published:** 2019-11-04

**Authors:** K. E. Rollins, N. C. Mathias, D. N. Lobo

**Affiliations:** ^1^ Gastrointestinal Surgery, Nottingham Digestive Diseases Centre, National Institute for Health Research Nottingham Biomedical Research Centre Nottingham University Hospitals and University of Nottingham, Queen's Medical Centre Nottingham NG7 2UH UK; ^2^ MRC Arthritis Research UK Centre for Musculoskeletal Ageing Research, School of Life Sciences University of Nottingham, Queen's Medical Centre Nottingham UK; ^3^ University of Exeter Medical School Exeter UK

We thank Dr Feldheiser and colleagues for their interest in our meta‐analysis examining the role of goal‐directed fluid therapy (GDFT) guided by transoesophageal Doppler in patients undergoing elective colorectal surgery^1^. We note their erroneous comment that the meta‐analysis^1^ was performed on RCTs that included patients undergoing abdominal surgery, and would like to reiterate that, although we have published a meta‐analysis on the role of GDFT in abdominal surgery previously^2^, the focus of the meta‐analysis under discussion^1^ was exclusively on the use of transoesophageal Doppler‐guided GDFT in elective colorectal surgery. This may explain their misunderstanding as to why the FEDORA study^3^ was excluded. The FEDORA study^3^ was an RCT that included 450 patients scheduled for major elective surgery, with patients undergoing abdominal, urological, gynaecological or orthopaedic surgery, although, owing to recruitment issues, the orthopaedic patients were excluded from the outcome analysis. The results^3^ did not consider postoperative outcomes separately for colorectal, or indeed abdominal, surgery alone, and as such the study did not meet our inclusion criteria^1^. The focus on elective colorectal surgery aimed to reduce the heterogeneity introduced by including different surgical populations^1^. In addition, as several guidelines^4^, ^5^ support the use of GDFT in elective colorectal surgery, we sought to establish the role of this technique in this study population alone^1^.

With regard to inclusion of the study by Brandstrup *et al*.^6^, this RCT compared GDFT with a zero‐balance fluid strategy. Our meta‐analysis^1^ aimed to compare those ‘randomized to receive either GDFT administered with transoesophageal Doppler monitoring or conventional intraoperative fluid therapy’. On review of the protocol for the zero‐balance group^6^, these patients received a slow infusion of 6 per cent hydroxyethyl starch to replace lost blood, an extra 500 ml to maintain the mean arterial pressure above 60 mmHg, and erythrocytes, plasma and thrombocytes when indicated. We therefore decided that this study was eligible for inclusion. This study^6^ has been included previously in a number of other meta‐analyses^7^, ^8^, ^9^, ^10^, ^11^ on the topic of intraoperative GDFT, so many other authors of meta‐analyses have agreed with this decision. In terms of the implication of exclusion of this study^6^, the results of the meta‐analysis^1^ are entirely unchanged and would still lack evidence to support the use of GDFT in elective colorectal surgery.

Thus, we feel that the study inclusion criteria for our meta‐analysis^1^ are correct and complete, and as such are confident in the conclusions. These findings^1^ are in line with the conclusions of our previous meta‐analysis^2^ conducted in elective major abdominal surgery, which showed a lack of benefit of GDFT compared with conventional intraoperative fluid therapy within enhanced recovery after surgery pathways, although a benefit was demonstrated when all studies using GDFT were considered. That is not to say that patients at high risk of complications, or those undergoing high‐risk surgical procedures, would not benefit from GDFT. However, there are insufficient data to conduct a synthesis of evidence on this aspect.

## Acknowledgements

D.N.L. has received unrestricted research funding from B. Braun and speaker's honoraria from Fresenius Kabi, B. Braun, Shire and Baxter Healthcare for unrelated work.

*Disclosure*: The authors declare no conflict of interest.
